# Genuine Dirac Half‐Metals in Two‐Dimensions

**DOI:** 10.1002/advs.202307297

**Published:** 2023-12-03

**Authors:** Jialin Gong, Guangqian Ding, Chengwu Xie, Wenhong Wang, Ying Liu, Gang Zhang, Xiaotian Wang

**Affiliations:** ^1^ Institute for Superconducting and Electronic Materials (ISEM) University of Wollongong Wollongong 2500 Australia; ^2^ School of Physical Science and Technology Southwest University Chongqing 400715 China; ^3^ School of Science Chongqing University of Posts and Telecommunications Chongqing 400065 China; ^4^ School of Electronics and Information Engineering Tiangong University Tianjin 300387 China; ^5^ School of Materials Science and Engineering Hebei University of Technology Tianjin 300130 China; ^6^ Institute of High Performance Computing Agency for Science Technology and Research (A*STAR) Singapore 138632 Singapore

**Keywords:** 100% spin polarization, 2D half‐metals, *d* ^0^ ferromagnetic materials, Dirac points

## Abstract

When spin‐orbit coupling (SOC) is absent, all proposed half‐metals with twofold degenerate nodal points at the K (or K′) point in 2D materials are classified as “Dirac half‐metals” owing to the way graphene is utilized in the earliest studies. Actually, each band crossing point at the K or K′ point is described by a 2D Weyl Hamiltonian with definite chirality; hence, it should be a Weyl point. To the best of its knowledge, there have not yet been any reports of a genuine (*i*.*e*., fourfold degenerate) 2D Dirac point half‐metal. In this work, using first‐principles calculations, it proposes *for* *the* *first* *time* that the 2D *d* ^0^‐type ferromagnet Mg_4_N_4_ is a genuine 2D Dirac half‐metal candidate with a fourfold degenerate Dirac point at the S high‐symmetry point, intrinsic magnetism, a high Curie temperature, 100% spin polarization, topology robust under the SOC and uniaxial and biaxial strains, and spin‐polarized edge states. This work can serve as a starting point for future predictions of intrinsically magnetic materials with genuine 2D Dirac points, which will aid the frontier of topo‐spintronics research in 2D systems.

## Graphical abstract

In this work, using first‐principles calculations, it proposes that the 2D d^0^‐type ferromagnet Mg_4_N_4_ is a 2D Dirac half‐metal candidate with a fourfold degenerate Dirac point at the S high‐symmetry point, intrinsic magnetism, a high Curie temperature, 100% spin polarization, topology robust under the SOC and uniaxial and biaxial strains, and spin‐polarized edge states.

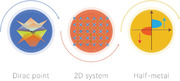

## 1 Introduction

The discovery of intrinsic magnetism in 2D crystals^[^ ^1^, ^2^, ^3^, ^4^, ^5^, ^6^, ^7^, ^8^, ^9^ ^]^ is a significant recent accomplishment in materials science, as it opens the door to new cutting‐edge disciplines in the 2D family and could lead to the development of revolutionary data storage and information systems with further downsizing.^[^ ^10^, ^11^, ^12^ ^]^ Several 2D ferromagnetic half‐metals, including 2D CrI_3_ monolayer, Cr_2_Ge_2_Te_6_ bilayer, and Fe_3_GeTe_2_ monolayer, have been theoretically predicted and actually synthesized.^[^ ^13^, ^14^, ^15^ ^]^ More interestingly, the link between magnetism and band topology in the 2D family has become a popular study topic.^[^ ^16^, ^17^, ^18^, ^19^, ^20^, ^21^, ^22^, ^23^, ^24^, ^25^, ^26^, ^27^, ^28^, ^29^, ^30^, ^31^, ^32^, ^33^, ^34^ ^]^ As indicated in Table S1 (Supporting Information), we reviewed a series of published literature and discovered that many researchers have proposed 2D half‐metals (or 2D spin‐gapless semiconductors) with linearly dispersed crossing points. We would like to indicate that all the linearly dispersed crossing points with twofold degeneracy proposed in the reviewed literature (see Table S1, Supporting Information) are classified as “Dirac points”.

This is probably because of how graphene was used in early research. Each band crossing point at the K or K′ in graphene is characterized by a 2D Weyl Hamiltonian with a definite chirality; hence, it should be a Weyl point. For concreteness, the effective model describing the band crossings at the K or K′ is expressed as

(1) $$H_{K/K^{\prime }}=\nu \left(\tau_{z}k_{x}\sigma_{x}+k_{y}\sigma_{y}\right)$$

where ν is the Fermi velocity and σ's is the Pauli matrix. Here, τ = ±1, which refers to the K and K′ valleys, respectively. Additionally, *k* is measured from the band crossings at the K/K′. According to the effective model, the eigenenergies for the crossing bands are $E_{c/\nu }=\pm \sqrt{k_{x}^{2}+k_{y}^{2}}$, which implies a double degeneracy at the K/K′ point. Hence, the degeneracy for the band crossings at the corners of the graphene Brillouin zone is twofold, rather than fourfold. Traditionally, the band crossings in graphene are referred to as Dirac points because the quasiparticles at these two valleys are combined into a single model. However, the degeneracy does not occur at the same point. Therefore, it is a 2D Weyl point. Note that the denomination we use are analogous to the massless spinor representation of the 3+1d Lorentz group. In other words, it is conceptually more coherent to refer to the aforementioned materials (listed in Table S1, Supporting Information) as Weyl half‐metals with Weyl points (twofold degenerate points that satisfy the Weyl model) rather than Dirac half‐metals with Dirac points (fourfold degenerate points that satisfy the Dirac model). Moreover, when spin‐orbit coupling (SOC) is considered, small gaps occur between two Weyl points (usually at the K and K′) in most of the proposed 2D Weyl half‐metals/spin‐gapless semiconductors. The opening of the gap for a 2D Weyl point may not be a negative thing; instead, it may result in intriguing physics, such as the quantum anomalous Hall effect in 2D systems.^[^ ^27^, ^35^ ^]^

As mentioned earlier, a Dirac point can be regarded to consist of two Weyl points with opposite chirality, *i*.*e*., one right‐handed Weyl point and one left‐handed Weyl point sitting together, making it fourfold degenerate. To date, it has been theoretically proposed that 2D Dirac points exist in 2D nonmagnetic semiconductors and the phonon curves of 2D materials.^[^ ^36^, ^37^, ^38^ ^]^ However, 2D Dirac points have not been proposed in magnetic candidates both theoretically and experimentally. Specifically, apart from 2D Weyl half‐metals/spin‐gapless semiconductors with twofold degenerate Weyl points around/at the Fermi level, no genuine 2D Dirac half‐metals/spin‐gapless semiconductors with fourfold degenerate Dirac points around/at the Fermi level can be found in the literature.

Possible reasons why 2D Dirac points have not been studied in 2D magnetic systems include the following: 1) In spintronics, linearly dispersed band crossing points in two dimensions are almost always referred to as Dirac points, regardless of the degeneracy of the bands surrounding the band crossing points.^[^ ^39^ ^]^ Consequently, the prediction of genuine 2D Dirac half‐metals has not received sufficient consideration. 2) There is still an ongoing search for intrinsic 2D magnetic materials, let alone a genuine 2D Dirac half‐metal with intrinsic magnetism and 100% spin polarization. 3) Additionally, 2D topological ferromagnets have two issues. The first^[^ ^26^, ^29^ ^]^ is that most topological signatures in 2D topological ferromagnets will be gapped under SOC. The other is that the current 2D topological ferromagnets have a relatively low Curie temperature. Therefore, finding genuine 2D Dirac half‐metals with a high Curie temperature and robustness to SOC may be difficult.

In this work, we propose Mg_4_N_4_, a 2D ferromagnetic material, as the first 2D Dirac half‐metal candidate with a fully spin‐polarized fourfold degenerate Dirac point at the S high‐symmetry point, a high Curie temperature of about 431 K, a visible fully spin‐polarized edge state, and robustness to SOC and uniaxial and biaxial strains. Moreover, the magnetic moments in existing 2D topological ferromagnets are primarily contributed by unoccupied *d*/*f* shells from transition‐metal or rare‐earth elements, a phenomenon known as *d*/*f*‐type ferromagnetism. However, the magnetism in Mg_4_N_4_ arises from the *p* orbitals. In other words, Mg_4_N_4_ is a 2D monolayer without unoccupied *d*/*f* shells, namely, a *d* ^0^ ferromagnet.^[^ ^18^, ^40^, ^41^, ^42^ ^]^ Moreover, *d* ^0^ ferromagnets have received less research than 2D *d*/*f*‐type ferromagnets, and they have unique properties, such as substantially weaker localization of *p* electrons and less SOC strength, which are significantly advantageous for high‐speed and long‐distance transport.^[^ ^40^, ^41^ ^]^

## 2 Results and Discussion

**Figure 1:**
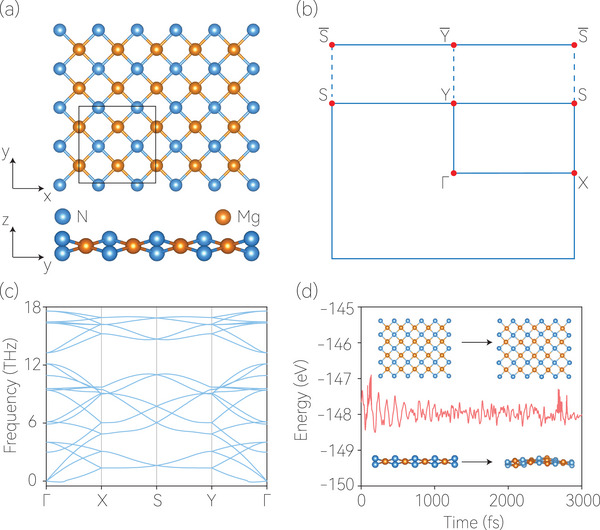
a) Top and side views of the Mg_4_N_4_ monolayer. The dashed box indicates the unit cell. b) The 2D Brillouin zone and its projection to the [010] edge. c) The calculated phonon dispersion for the 2D Mg_4_N_4_ unit cell. To obtain the force constants, we used a 2 × 2 × 1 Mg_4_N_4_ supercell. d) Ab initio molecular dynamics of Mg_4_N_4_ at 300 K.

Herein, we select 2D Mg_4_N_4_ with layer group (LG) No. 45 (space group No. 57) as a typical example. The lattice structure of the Mg_4_N_4_ monolayer is displayed in **Figure** **1**a. The lattice parameters of Mg_4_N_4_ are a = 5.7806 Å and b = 5.7815 Å. More details on the atomic positions of Mg_4_N_4_ are provided in Table S2 (Supporting Information). It is necessary to confirm the dynamical and thermal stability of the lattice before investigating its electronic band structures. Therefore, we used the density functional perturbation theory^[^ ^43^ ^]^ to obtain the force constants, as implemented in the VASP. Thereafter, we used the PHONOPY package^[^ ^44^ ^]^ to calculate the phonon dispersion spectrum. Figure 1c shows no imaginary frequency in the phonon spectrum, indicating that 2D Mg_4_N_4_ is dynamically stable. We performed *ab* *initio* molecular dynamics calculations^[^ ^45^ ^]^ in a 2 × 2 × 1 supercell to estimate the thermal stability of 2D Mg_4_N_4_. As shown in Figure 1d, after 3000 steps at 300 K, we discovered that the final states exhibited thermal‐induced fluctuations without bond breakage or geometric reconfigurations, demonstrating that 2D Mg_4_N_4_ is thermally stable at room temperature. Mg_4_N_4_ monolayer has not been synthesized, but we may use the following methods to synthesize it: i) One may use Mg as substrate and N_2_ gas as the nitrogen source by Chemical vapor deposition to obtain the monolayer. 2) One may grow the Mg_4_N_4_ film on a MgO substrate by molecular beam epitaxy.

**Figure 2:**
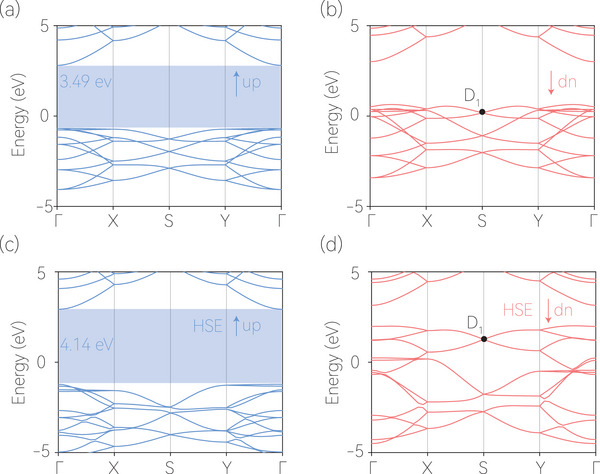
a) Spin‐up and b) spin‐down band structures of 2D Mg_4_N_4_ calculated using the GGA method. c) Spin‐up and d) spin‐down band structures of 2D Mg_4_N_4_ calculated using the HSE 06 method. D_1_ indicates the fully spin‐polarized Dirac point in the spin‐down channel.

Note that 2D Mg_4_N_4_ is a *d* ^0^ ferromagnet without unoccupied *d*/*f* shells. In our calculations, we considered four possible magnetic configurations for the unit cell: one nonmagnetic, one ferromagnetic, and two antiferromagnetic states (see Figure S1, Supporting Information). We compared their energies to identify the ground magnetic state of the Mg_4_N_4_ monolayer and discovered that the ferromagnetic state has the lowest energy and should be the ground magnetic state for 2D Mg_4_N_4_. Additionally, six magnetic configurations in the 2 × 2 × 1 supercell, comprising one nonmagnetic, one ferromagnetic, and four antiferromagnetic states, were investigated to confirm the magnetic ground state of 2D Mg_4_N_4_ (see Figure S2, Supporting Information). Evidently, the ferromagnetic state for Mg_4_N_4_ remains the lowest magnetic state. Table S3 (Supporting Information) shows the determined total and atomic magnetic moments for 2D Mg_4_N_4_. Table S3 (Supporting Information) reveals that practically all magnetic moments are concentrated around the N atoms, indicating that the magnetic character of Mg_4_N_4_ is primarily due to the N atoms. As the Pauling electronegativity of the N atom (3.04) is much larger than that of the Mg atom (1.31), the four Mg atoms donate eight electrons to the four N atoms within the Mg_4_N_4_ unit cell. Namely, the four N atoms would completely hold 20 valence electrons by sharing eight additional Mg‐3*s* electrons. According to Hund's rule, four N atoms should exhibit an electronic configuration of (↑↓↑↓↑↓↑↓↑↓↑↓↑↓↑↓↑↑↑↑), leaving four unpaired electrons (see Figure S3, Supporting Information), with each N atom having an atomic magnetic moment of 1μ_*B*_. As shown in Table S3 (Supporting Information), the calculated atomic magnetic moment for each N in Mg_4_N_4_ is 0.65 μ_*B*_. The atomic magnetic moment of N was also observed in other 2D *d* ^0^ ferromagnets,^[^ ^46^, ^47^ ^]^ such as 2D T‐K_2_N with an atomic magnetic moment of 0.721 μ_*B*_ per N, and 2D FE‐ZB' Tl_2_NO_2_ with an atomic magnetic moment of 0.61 μ_*B*_ per N, and 2D FE‐ZB' In_2_NO_2_ with an atomic magnetic moment of 0.59 μ_*B*_ per N. The projected density of states depicted in Figure S4b, (Supporting Information) shows that the low‐energy bands are mainly from the N‐*p* orbital, confirming that the Mg_4_N_4_ monolayer is a typical *d* ^0^ ferromagnet. Additionally, we plot the electronic band structure of 2D Mg_4_N_4_ without considering SOC in **Figure** **2**. The band structures obtained from the GGA^[^ ^48^ ^]^ in the spin‐up and spin‐down channels are shown in Figure 2a,b, respectively. Moreover, the Heyd‐Scuseria‐Ernzerhof (HSE06) hybrid functional,^[^ ^49^ ^]^ which is more accurate but computationally expensive, was employed to double‐check the spin‐polarized band structures of 2D Mg_4_N_4_, as exhibited in Figure 2c,d. It can be observed that the spin‐up and spin‐down states are separated, with the latter contributing primarily to the states at the Fermi level. The spin‐polarization ratio can be calculated using the following formula:^[^ ^50^, ^51^ ^]^

(2) $$P\left(\%\right)=\frac{N↑\left(E_{F}\right)-N↓\left(E_{F}\right)}{N↑\left(E_{F}\right)+N↓\left(E_{F}\right)}$$

where N↑(*E*_*F*_) and N↓(*E*_*F*_) are the spin‐up and spin‐down electrons at the Fermi level (*E*_*F*_), respectively. Since N↑(*E*_*F*_) = 0, 2D Mg_4_N_4_ should be a half‐metal with 100% spin polarization around the Fermi level.

Near the Fermi level, it can be observed that the two bands (from the spin‐down channel) along the X‐S and Y‐S high‐symmetry paths exhibit twofold degeneracy. These two doubly degenerate bands along the X‐S and Y‐S high‐symmetry paths belong to Weyl nodal lines, which can be understood from the following symmetry analysis. LG 45 is generated by two screw rotation symmetries, namely, $S_{2x}:(x,y,z)\to \left(x+\frac{1}{2},-y+\frac{1}{2},-z\right)$ and $S_{2y}:(x,y,z)\to \left(-x,y+\frac{1}{2},-z\right)$ and inversion symmetry P. In the absence of SOC, the two spin channels are decoupled, and each spin state could be regarded as a spinless system such that the original symmetry in a nonmagnetic system, including the time‐reversal symmetry T, is preserved in each spin channel. Since the states on the X‐S path are invariant under the combined Kramer‐like operation $TS_{2x}$, one can also derive that ${\left(TS_{2x}\right)}^{2}=e^{-\mathrm{ikx}}=e^{-i\pi }=-1$ on this path. Hence, any band on this path is at least doubly degenerate because of this Kramer‐like operation. Moreover, the Y‐S path is the common invariant subspace for $TS_{2y}$, with ${\left(TS_{2y}\right)}^{2}=e^{-\mathrm{iky}}=e^{-i\pi }=-1$ on this path, which results in a double degeneracy at any *k* point on this path. Therefore, each band on these two paths is at least doubly degenerate, resulting in nodal lines along these two boundaries of the Brillouin zone.

**Figure 3:**
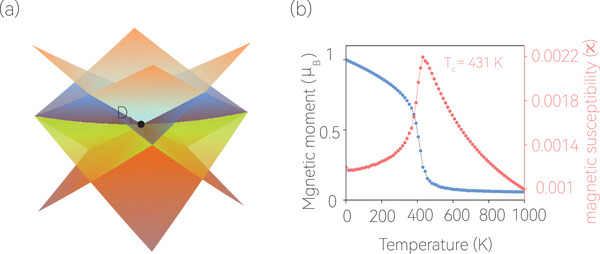
a) The 3D plot of the four bands around the D_1_ Dirac point at the S high‐symmetry point. b) The dependence of magnetic moment and magnetic susceptibility on the temperature demonstrated by the Heisenberg model via Monte Carlo simulations.

Moreover, the two doubly degenerate bands overlap with each other and form a fourfold degenerate point (D_1_) at the S point. Actually, such a fourfold degenerate D_1_ point is a 2D Dirac point that arises solely from the spin‐down state (Figure 2b,d), making 2D Mg_4_N_4_ the first proposed genuine 2D Dirac half‐metal candidate with 100% spin polarization. For clarity, the 3D plot of the spin‐down Dirac point with fourfold degeneracy is shown in **Figure** **3**a. We also use symmetry analysis to further comprehend the nature of the fourfold degenerate Dirac point at the S high‐symmetry point as follows. We previously mentioned that any point on the Y‐S path, including the S point, exhibits double degeneracy because of the Kramer‐like operation $TS_{2y}$. We also note that the S point is an invariant point under inversion symmetry P. Additionally, $PS_{2y}$ = $e^{-ik_{y}}S_{2y}P$, which implies that these two operations anticommute at the S (π, π) point, with $\left\{P,\;S_{2y}\right\}$ = 0. Therefore, the states at the S point are fourfold degenerate.

Next, we come to further understand the physics of the Dirac point in the spin‐down channel of 2D Mg_4_N_4_. If SOC is not considered, the spin‐up and spin‐down bands have no symmetry connecting them, making it possible to view the bands for each spin direction as a spinless system. Hence, we could choose a simple spinless four‐band lattice model to demonstrate the key characteristics of the 2D Dirac point in LG 45.

A unit cell with one site (0,0,0) was considered in a spinless lattice model with LG 45, and the *p*_*x*_ orbital was placed on this site. The spinless four‐band lattice model can be written as

(3) $$\left(\begin{array}{cccc} H_{11} & H_{12} & 0 & H_{14}\\ & H_{11} & H_{14} & 0\\ & & H_{11} & H_{12}\\ † & & & H_{11} \end{array}\right)$$

where $H_{11}=2r_{2}\cos\left(k_{z}\right)$, $H_{12}=2t_{1}\cos\left(\frac{k_{z}}{2}\right)$, and $H_{14}=2r_{1}\cos\left(\frac{k_{x}}{2}\right)$. Figure S5 (Supporting Information) depicts the band structure of the spinless lattice model (see Equation (3)) along the Γ‐X‐S‐Y‐Γ paths. We set *e*_1_ = 0, *r*_1_ = ‐0.115, *r*_2_ = 0.005, and *t*_1_ = ‐0.125 for the bands, and a fourfold Dirac point appeared at the S high‐symmetry point. This simple model may serve as a starting point for future research into the 2D Dirac point in LG 45.

Different from the 2D Weyl points in graphene, which can be characterized by a quantized Berry phase, the Berry phase associated with the Dirac point in this system is zero, which can be understood based on its effective model. Based on the TB model shown in Equation (3), we expand it up to the first‐order of *k* at the S point. In order to easily get its feature, we show the effective model in a standard form after a unitary transformation, specifically,

(4) $$\begin{array}{c} H_{k\cdot p}=\left(\begin{array}{cccc} 0 & t_{1}q_{z}+r_{1}q_{x} & 0 & 0\\ t_{1}q_{z}+r_{1}q_{x} & 0 & 0 & 0\\ 0 & 0 & 0 & t_{1}q_{z}-r_{1}q_{x}\\ 0 & 0 & t_{1}q_{z}-r_{1}q_{x} & 0 \end{array}\right)=\left(\begin{array}{cc} h_{+} & 0\\ 0 & h_{-} \end{array}\right) \end{array}$$

here, ***q*** is measured from the band crossing at the S point. We neglect the term linear to identity matrix since it does not affect the topology. Then, one could regard this Dirac point as a composition of two models described by the following 2 × 2 matrixes,

(5) $$h_{\pm }=\left(t_{1}q_{z}\pm r_{1}q_{x}\right)\sigma_{x}.$$

Since these two models are only dependent of $\sigma_{x}$, their eigenstates |*u*〉 are independent of q, resulting in a vanishing Berry connection (A), which is defined as $A=i\langle u|\nabla_{q}|u\rangle$. Consequently, the Berry phase for them also vanishes. Thus, the reported Dirac point has a zero Berry phase.

The Curie temperature determines the thermal stability of the ferromagnetic ordering and is critical for spintronic device applications. Therefore, we examined the Curie temperature of 2D Mg_4_N_4_ via Monte Carlo simulations using the Heisenberg model incorporated with single‐ion anisotropy.^[^ ^52^ ^]^ Additional details on the simulations can be found in the Supporting Information. The magnetic moment per unit cell and the magnetic susceptibility are shown in Figure 3(b), which shows that the Curie temperature of the 2D Mg_4_N_4_ monolayer is as high as 431 K.

**Figure 4:**
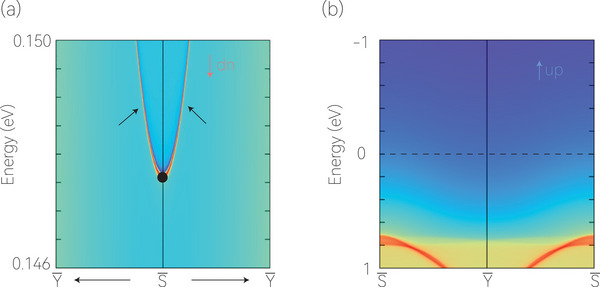
The [010] projective spectra in the a) spin‐down and b) spin‐up channels. The fully spin‐polarized edge states connecting to the projection of the D_1_ Driac point are apparent in (a).

Furthermore, we studied the edge states of the Dirac point around the Fermi level for the [010] projective spectra (see Figure 1b). The results are shown in **Figure** **4**. The location of the projected Dirac point in the spin‐down channel is indicated by a black ball. The edge states are clearly connected by the projected Dirac point. Note that the edge states only appear in the spin‐down channel, indicating the fully spin‐polarized nature. Such clean spin‐polarized edge states will be advantageous for follow‐up experiments using surface‐sensitive sensors, such as electron energy loss spectroscopy and helium scattering.

Before concluding this work, we would like to discuss the following: 1) A trend in recent 2D research is to push the study of topological phases to magnetic systems, and then to unveil unique phenomena resulting from the combination of band topology and magnetism. The Dirac half‐metals in 2D systems have never been reported, and thus, Mg_4_N studied in this work is the first proposed genuine 2D Dirac half‐metal candidate with 100% spin polarization. Notably, compared to 3D materials, 2D materials with less symmetrical constraints may more intuitively display the characteristics of Dirac points. Our work considerably broadens the candidate range of Dirac half‐metals in 2D systems and opens up a new direction for topo‐spintronics in 2D systems. 2) We further investigated the robustness of the Dirac point in the 2D Mg_4_N_4_ monolayer to the effects of SOC and uniaxial and biaxial strains (see the details in Figures S6–S8, Supporting Information). The results revealed that the Dirac point is robust to the SOC, uniaxial strain, and biaxial strain for the 2D Mg_4_N_4_ monolayer. Note that we set the magnet direction along the z axis. Then, the combined operation $TS_{2x}$ still persists, and the inversion symmetry and $S_{2z}$ are preserved. When considering SOC, we still have ${\left(TS_{2x}\right)}^{2}=-1$ on path X‐S, such that any band on this path is at least double degenerate. In addition, we have $PS_{z}$ = $e^{-ik_{x}}S_{z}P$. So, we have $\left\{P,\;S_{2z}\right\}$ = 0 at the S point, also suggesting a double degeneracy at this point. Given all of symmetries, the crossing point in the presence of SOC at the S point is a 2D fourfold degenerate Dirac point. Consequently, the Dirac point at the S point in Mg_4_N_4_ is robust against SOC. 3) Apart from 2D Mg_4_N_4_ with LG 45, 2D Na_4_O_4_ with LG 33 is also used as an example to demonstrate that it is a Dirac half‐metal candidate with a fourfold degenerate Dirac point at the S high‐symmetry point and with intrinsic magnetism, a high Curie temperature, 100% spin polarization, and robustness to the uniaxial and biaxial strains (see the details in Figure S9–S17, Supporting Information). Moreover, as shown in Figure S13 (Supporting Information), we considered a unit cell with one site (0,0,0) and placed the *p*_*x*_ orbital on the (0,0,0) site. Subsequently, we built a spinless four‐band lattice model to demonstrate the nature of the 2D Dirac point in LG 33 using Equation (3), where $H_{11}=e_{1}+2r_{2}\cos\left(k_{z}\right)$, $H_{12}=2r_{1}\cos\left(\frac{k_{z}}{2}\right)$, and $H_{14}=2t_{1}\cos\left(\frac{k_{x}}{2}\right)$.

## 3 Conclusion

In summary, we theoretically propose that 2D *d* ^0^ ferromagnet Mg_4_N_4_ (with LG 45), a dynamically and thermodynamically stable material, is the first mentioned genuine 2D Dirac half‐metal candidate with a fourfold degenerate Dirac point at the S high‐symmetry point, as well as intrinsic magnetism, a high Curie temperature, 100% spin polarization, and robustness to SOC and uniaxial and biaxial strains. Similar physics is also exhibited by 2D *d* ^0^ ferromagnet Na_4_O_4_ (with LG 33). In addition to proposing the first genuine 2D *d* ^0^ Dirac half‐metal candidate, this work also offers simple models that demonstrate the key characteristics of the 2D Dirac points in LGs 45 and 33, which can be viewed as a starting point for predicting genuine 2D Dirac half‐metals in both LGs.

## 4 Experimental Section

The first‐principles calculations in the framework of density‐functional theory (DFT) was performed within the Vienna Ab initio simulation package (VASP).^[^ ^53^, ^54^ ^]^ The exchange‐correlation effect was treated in the generalized gradient approximation (GGA) in the Perdew Burke Ernzerhof (PBE) function.^[^ ^48^ ^]^ A vacuum layer of 20 Å thick was used to ensure decoupling between neighboring slabs. All calculations were performed with a plane‐wave cutoff of 500 eV, and the convergence criterion for the electronic self‐consistence loop was set to be 10^−6^ eV on the 9 × 9 × 1 Monkhorst‐Pack k‐point mesh, and for the structural relaxation, the Hellmanne‐Feynman forces on each atom were taken to be ‐0.01 eV Å^‐1^. VESTA package was used for the illustration of atomic structures.^[^ ^55^ ^]^ The topological features of edge states were calculated based on maximally localized Wannier functions,^[^ ^56^ ^]^ realized using the WANNIERTOOLS package.^[^ ^57^ ^]^ It should be noted that the more accurate but computationally expensive Heyd‐Scuseria‐Ernzerhof (HSE06) hybrid functional^[^ ^49^ ^]^ was employed to calculate the electronic structure. The phonon dispersion calculations were fulfilled by the density functional perturbation theory^[^ ^58^ ^]^ coded in the PHONOPY package.^[^ ^44^ ^]^ Ab initio molecular dynamics (AIMD) simulations last for 3.0 ps with a time step of 1.0 fs in the canonical ensemble with the temperature fixed at 300 K by the Nose‐Hoover thermostat.^[^ ^59^ ^]^

## Conflict of Interest

The authors declare no conflict of interest.

## Supporting information

Supporting Information

## Data Availability

The data that support the findings of this study are available from the corresponding author upon reasonable request.
