# Supplementary material for: Genuine Dirac Half‐Metals in Two‐Dimensions
Source: Adv Sci (Weinh). 2023 Dec 3;11(6):2307297. doi: 10.1002/advs.202307297 (PMC10853703; doi:10.1002/advs.202307297)
Supplement: Supplementary file 1 — Supporting Information [file ADVS-11-2307297-s001.pdf]

ADVANCED SCIENCE
Open Access

Supporting Information

for *Adv. Sci.*, DOI 10.1002/advs.202307297

Genuine Dirac Half-Metals in Two-Dimensions

Jialin Gong, Guangqian Ding, Chengwu Xie, Wenhong Wang, Ying Liu, Gang Zhang* and Xiaotian Wang**

TABLE S1. Previously proposed 2D Weyl half-metals.

Materials	Ref.	Materials	Ref.	Materials	Ref.
$Mn(C_6H_5)_3$	[1]	1T-LaN_2	[2]	h-V_2O_3	[3]
1T-CrO_2	[4]	Mn-Pc	[5]	CrO_2/TiO_2	[6]
Cd_2N_3	[7]	h-VN	[8]	YN_2	[9]
$Mn_2C_6S_{12}$	[10]	MnX_3 (X = F, Cl, Br, I)	[11]	CrB_2C_2	[12]
$NiBr_3$, NiI_3	[13]	CoB_6	[14]	MnNF	[15]
Cr_2X_3 (X = S; Se; Te)	[16]	OsI_3	[17]	Na_3Te_2	[18]
$Ni_2C_{18}H_{12}$ and $Co_2C_{18}H_{12}$	[19]	Na_2C	[20]	$PdCl_3$	[21]
Zn_3Si_2	[22]	VCl_3, VI_3	[23]	C_3Ca_2	[24]
$NiCl_3$	[25]	$M_3C_{12}S_{12}$ and $M_3C_{12}O_{12}$ (M = Zn, Cd, Hg, Be, or Mg)	[26]	$Ni_2C_{24}S_6H_{12}$	[27]
1T-$RemN_2$	[28]	$Mn_2C_6Se_{12}$ and $Mn_2C_6S_6Se_6$	[29]	$Mn_2C_6S_{12}$	[30]
ReI_3, $ReBr_3$	[31]	Ta_2S_3	[32]	V-SF and Ti-SF	[33]
$TM_2(C_2O_4)_3$	[34]				

S1. 2D Weyl half-metals those labeled as 2D "Dirac" half-metals

In literature, linearly dispersing band crossing points in 2D are almost invariably called "Dirac points". This probably stems from the usage in the early works on graphene. Especially, without spin-orbit coupling (SOC), many 2D half-metals with twofold degenerate Weyl points were labeled as 2D Dirac half-metals instead of 2D Weyl half-metals. We listed 34 references (see Refs.[1–34]), in which the twofold degenerate Weyl points were labeled as Dirac points. When SOC is included, a sizable bulk band gap opens at the twofold degenerate points around the Fermi level in the listed 2D d/f-type ferromagnets in Table S1.

S2. Curie temperature calculations

The spin Hamiltonian, including the magnetic anisotropy term, is defined as follows[45–47]:

$$H = E_0 - \sum_{i,j} J_1 S_i \cdot S_j - \sum_{i,k} J_2 S_i \cdot S_k - \Sigma_i A \left(S_i^z\right)^2$$

J_1 and J_2 are the nearest neighboring exchange interaction parameter and the next nearest neighboring exchange interaction parameter, respectively. A is the single-ion anisotropic energy parameter, *i.e.*, $A = \frac{E[100]-E[001]}{|S|^2}$ [46].

Without spin-orbital coupling, the J_1 and J_2 can be found by mapping Hamiltonians on three magnetic configurations.

$$E_{FM} = E_0 - 8J_1S^2 - 8J_2S^2,$$
$$E_{AFM1} = E_0 - 8J_2S^2,$$
$$E_{AFM2} = E_0 + 8J_1S^2 - 8J_2S^2.$$

We found that the relative energies of AFM1 and AFM2 configurations with respect to the FM one are 0.373 eV and 0.607 eV, respectively, suggesting the FM ground state. Moreover, spin (S) is approximately set as 1 owing to the magnetic moment 0.65 μ_B of each N atom. Therefore, we extract $J_1 = 37.94$ meV and $J_2 = 4.35$ meV, respectively, for 2D Mg_4N_4. Using the derived J_1 and J_2, we can perform the statistical Monte Carlo (MC) simulation to estimate T_c via the mcsolver package[48–50].

To further confirm the reliability of our MC simulations, we also calculated the T_c values of synthesized CrX_3(X = Cl, Br, and I) monolayers. The values are listed in Table S4, which agree with the experimentally measured values[51].

S3. Effect of spin-orbit coupling

In order to analyze the change of the Dirac point under the spin-orbit coupling (SOC) effect, we have studied the SOC effect on 2D Mg_4N_4. Table S5 shows that Mg_4N_4 prefers the ferromagnetic state with the spin aligned along the [001] direction in the unit cell under SOC. To display this directly, we calculate the band structure of 2D Mg_4N_4 with SOC effect using the first-principles method. The results are shown in Figures S6(a) and S6(b). As the electronic states around the Fermi energy level of Mg_4N_4 are mainly contributed by the p orbitals of N atoms (see Figure S4), the SOC effect can be ignored. We artificially increase the impact of SOC by 10 times, and then calculate the band structure of 2D Mg_4N_4. The obtained results are presented in Figures S6(c) and S6(d). The Dirac point

D_1 is maintained, reflecting the negligibility of the SOC effect.

S4. Effects of uniaxial and biaxial strains

To assure the presence of a fully spin-polarized Dirac point, two conditions must be met: first, the spin-up channel must have a band gap, which enables the half-metallic character; second, the spin-down channel must have a fourfold band crossing point around the Fermi level, which ensure the presence of a Dirac point.

To show the robustness of the spin-polarized Dirac point in 2D Mg_4N_4, we have examined the electronic band structure against the uniaxial and biaxial strains.

Figures S7(a) and S8(a) show the positions at the bottom of the conduction band and the top of the valence band versus the -5%-5% uniaxial strains/biaxial strains. We can see that the band gap between the conduction band and the valence band in the spin-down channel always exists under -5%-5% uniaxial strains/biaxial strains. For the spin-up channel, we show the energy positions of fourfold degenerate point D_1 versus the -5 to +5% uniaxial strains/biaxial strains (see Figures S7(b) and S8(b)). We find that such fourfold degenerate point D_1 always remains during the shift of uniaxial strains/biaxial strains. Hence, the fully spin-polarized Dirac point maintains during the change of uniaxial strains/biaxial strains. As examples, the spin-polarized band structures under -5% uniaxial strain, +5% uniaxial strain, -5% biaxial strain, and +5% biaxial strain are given in Figures S7(c), S7(d), S8(c), and S8(d), respectively. These results suggest that the fully spin-polarized Dirac point in 2D Mg_4N_4 is very robust to the strains, which may be meaningful for its future detection in experiments.

S5. 2D Dirac half-metal Na_4O_4 with LG 33

We also proposed that 2D Na_4O_4 is a 2D half-metal ferromagnet with a spin-polarized Dirac point around the Fermi level. The atomic positions for the LG 33 Na_4O_4 are shown in Table S6 and Figure S9. The unit cell of Na_4O_4 contains 4 Na and 4 O atoms, respectively. The lattice parameters of Na_4O_4 are a = 4.692 Å and b = 4.690 Å. According to Table S7, the magnetic moment is found to be 3.24 μ_B per formula unit, mainly attributed to the O atoms. To determine the magnetic ground state of Na_4O_4, an energy comparison was made among three different configurations: FM, AFM as well as the NM systems. Different magnetic configurations in the $1 \times 1 \times 1$ unit cell and $2 \times 2 \times 1$ supercell are presented in Figures S10 and S11, respectively. One finds that the optimized FM state is most energetically stable in all magnetic configurations and has lower energy than the AFM and NM states, respectively.

Having identified the magnetic ground state of Na_4O_4, the detailed spin-polarized band structures are shown in Figures S12(a) and S12(b). Without SOC, one finds a fourfold Dirac point (D_2) above the Fermi level in the spin-down channel. Only spin-down O-p orbitals contribute to the D_2 point (see Figure S12(d)), and a 5.03 eV energy gap appears in the spin-up channel. Hence, the Dirac point D_2 is 100% spin-polarized.

Based on the unit cell of 2D Na_4O_4, one finds that relative energies of AFM1 and AFM2 configurations with respect to the FM one are 0.340 eV and 0.467 eV, respectively, suggesting the FM ground state. Moreover, spin S is approximately set as 1 owing to the magnetic moment 0.81 μ_B of each O atom. Therefore, we extract $J_1 = 29.18$ meV and $J_2 = 6.64$ meV, respectively. Using the derived J_1 and J_2, we can perform the statistical MC simulation to estimate T_c via the mcsolver package[48–50]. The simulated result is shown in Figure S14(b), from which one observes $T_c = 362$ K. The high-temperature ferromagnetism makes the 2D Na_4O_4 monolayer promising for practical spintronic applications.

In addition, we investigated the [100] edge states of the Dirac point D_2. Figure S15 shows the calculated results. A black ball indicates the location of the projected Dirac point in the spin-down channel. Obviously, the projected Dirac point connects the edge states. We note that edge states only exist in the spin-down channel, which is fully spin-polarized.

Moreover, as shown in Figures S16 and S17, the fully spin-polarized Dirac point in 2D Na_4O_4 is very robust to the uniaxial and biaxial strains, which may be meaningful for its future detection in experiments.

---

[1] Z. Wang, Z. Liu, and F. Liu, Phys. Rev. Lett. 110, 196801 (2013).
[2] L. Li, X. Kong, X. Chen, J. Li, B. Sanyal, and F. M. Peeters, Appl. Phys. Lett. 117, 143101 (2020).
[3] H. van Gog, W.-F. Li, C. Fang, R. S. Koster, M. Dijkstra, and M. van Huis, npj 2D Mater. Appl. 3, 18 (2019).
[4] S. He, P. Zhou, Y. Yang, W. Wang, and L. Z. Sun, Nanoscale Adv. 3, 3093 (2021).
[5] I. Choudhuri, P. Bhauriyal, and B. Pathak, Chem. Mater. 31, 8260 (2019).
[6] T. Cai, X. Li, F. Wang, S. Ju, J. Feng, and C. D. Gong, Nano Lett. 15, 6434 (2015).
[7] X. Y. Li, W. X. Ji, P. J. Wang, and C. W. Zhang, Nanoscale Adv. 3, 847 (2021).
[8] A. V. Kuklin, S. A. Shostak, and A. A. Kuzubov, J. Phys. Chem. Lett. 9, 1422 (2018).
[9] Z. F. Liu, J. Y. Liu, and J. J. Zhao, Nano Res. 10, 1972 (2017).

[10] A. Wang, X. Zhang, Y. Feng, and M. Zhao, J. Phys. Chem. Lett. 8, 3770 (2017).
[11] Q. Sun, and N. Kioussis, Phys. Rev. B 97, 094408 (2018).
[12] B. W. Zhang, X. J. Chen, and J. Wang, Appl. Phys. Lett. 119, 162401 (2021).
[13] Z. Li, B.-Z Zhou., C.-B. Luan, RSC Adv. 9, 35614 (2019).
[14] X. Tang, W. Sun, Y. Gu, C. Lu, L. Kou, and C. Chen, Phys. Rev. B 99, 045445 (2019).
[15] Y. Hu, S.-S. Li, W.-X. Ji, C.-W. Zhang, M. Ding, P.-J. Wang, and S.-S. Yan, J. Phys. Chem. Lett. 11, 485 (2020).
[16] Y.-L. Feng, N. Liu, and G.-Y. Gao, Appl. Phys. Lett. 111, 202405 (2017).
[17] X. T. Fang, B. Z. Zhou, X. C. Wang, and W. B. Mi, Mater. Today Phys. 28, 100847 (2022).
[18] Y. C. Zhao, M. X. Zhu, Y. Wang, and P. Li, Chem. Phys. 562, 111658 (2022).
[19] Y. Ma, Y. Dai, X. Li, Q. Sun, and B. Huang, Carbon 73, 382 (2014).
[20] W. X. Ji, B. M. Zhang, S. F. Zhang, C. W. Zhang, M. Ding, P.-J. Wang, and R. Zhang, Nanoscale 10, 13645 (2018).
[21] Y. P. Wang, S. Li, C. W. Zhang, S. F. Zhang, W. Ji, P. Li, and P. Wang, J. Mater. Chem. C 6, 10284 (2018).
[22] Y. R. Guan, L. L. Song, H. Zhao, R. J. Du, L. M. Liu, C. X. Yan, and J. M. Cai, Chinese Phys. B 29, 087103 (2020).
[23] J. He, S. Ma, P. Lyu, and P. Nachtigall, J. Mater. Chem. C 4, 2518 (2016).
[24] W. X. Ji, B. M. Zhang, S. F. Zhang, C. W. Zhang, M. Ding, P. Li, and P.-J. Wang, J. Mater. Chem. C 5, 8504 (2017).
[25] J. He, X. Li, P. Lyu, and P. Nachtigall, Nanoscale 9, 2246 (2017).
[26] M. Wu, Z. Wang, J. Liu, W. Li, H. Fu, L. Sun, X. Liu, M. Pan, H. Weng, and M. Dinc, 2D Mater. 4, 015015 (2017).
[27] L. Wei, X. Zhang, and M. Zhao, Phys. Chem. Chem. Phys. 18, 8059 (2016).
[28] Y. Yu, X. Chen, X. Liu, J. Li, B. Sanyal, X. Kong, F. M. Peeters, and L. Li, Phys. Rev. B 105, 024407 (2022).
[29] X. Wu, Y. Feng, S. Li, B. Zhang, and G. Gao, J. Phys. Chem. C 124, 16127 (2020).
[30] A. Wang, X. Zhang, Y. Feng, and M. Zhao, J. Phys. Chem. Lett. 8, 3770 (2017).
[31] Q. Sun and N. Kioussis, Nanoscale 11, 6101 (2019).
[32] L. Zhang, C.-W. Zhang, S.-F. Zhang, W.-X. Ji, P. Li, and P.-J. Wang, Nanoscale 11, 5666 (2019).
[33] C. Tang, C. M. Zhang, Z. Y. Jiang, K. Ostritov, and A. Du, J. Mater. Chem. C 7, 5792, (2019).
[34] J. Xing, X. Jiang, Z. Liu, Y. Qi, and J. Zhao, Nanoscale 14, 2023 (2022).
[35] G. Kresse and J. Hafner, Phys. Rev. B 49, 14251 (1994).
[36] G. Kresse and J. Furthmller, Phys. Rev. B 54, 11169 (1996).
[37] J. P. Perdew, K. Burke, and M. Ernzerhof, Phys. Rev. Lett. 77, 3865 (1996).
[38] K. Momma and F. Izumi, J. Appl. Cryst. 41, 653 (2008).
[39] A. A. Mostofi, J. R. Yates, Y.-S. Lee, I. Souza, D. Vanderbilt, and N. Marzari, Comput. Phys. Commun. 178, 685 (2008).
[40] Q. S. Wu, S. N. Zhang, H. F. Song, M. Troyer, and A. A. Soluyanov, Comput. Phys. Commun. 224, 405 (2018).
[41] J. Heyd, G. E. Scuseria, and M. Ernzerhof, J. Chem. Phys. 118, 8207 (2003).
[42] P. Giannozzi and S. Baroni, Density-Functional Perturbation Theory, in Handbook of Materials Modeling: Methods, ed. S. Yip, Springer Netherlands, Dordrecht, pp. 195-214 (2005).
[43] A. Togo and I. Tanaka, Scr. Mater. 108, 1 (2015).
[44] D. Bucher, L. C. T. Pierce, J. A. McCammon, and P. R. L. Markwick, J. Chem. Theory Comput. 7, 890 (2011).
[45] G. S. Joyce, Phys. Rev. 115, 478 (1967).
[46] B. Wang, Q. S. Wu, Y. H. Zhang, Y. L. Guo, X. W. Zhang, Q. H. Zhou, S. Dong, and J. L. Wang, Nanoscale Horiz. 3, 551 (2018).
[47] B. Wang, X. Zhang, Y. Zhang, S. Yuan, Y. Guo, S. Dong, and J. Wang, Mater. Horiz. 7, 1623 (2020).
[48] L. Liu, X. Ren, J. H. Xie, B. Cheng, W. K. Liu, T. Y. An, H. W. Qin, and J. F. Hu, Appl. Surf. Sci. 480, 300 (2019).
[49] W. Li, H. Hu, and J. H. Choi, Phys. Rev. B **1**03, 195421 (2021).
[50] J. J. Shi, W. W. Ren, X. Y. Ren, Y. Y. Shang, R. Pang, and S. F. Li, J. Mater. Chem. A 10, 17766 (2022).
[51] H. H. Kim, B. Yang, and S. Li et al., Proc. Natl. Acad. Sci. U.S.A. 116, 11131 (2019).

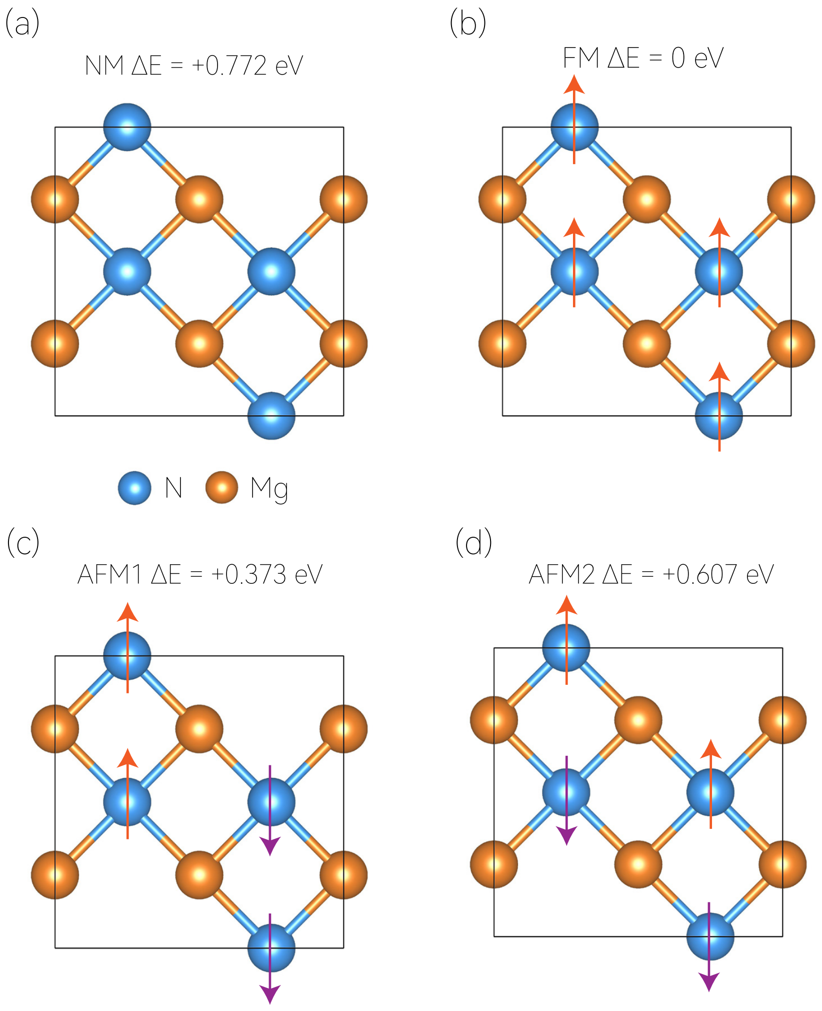

FIG. S1. Relative energies for (a) nonmagnetic (NM), (b) ferromagnetic (FM), and (c) and (d) two antiferromagnetic (AFM) states in a unit cell of Mg_4N_4. The arrows only indicate the spin directions of the N atoms. Key: bule, N atoms; orange, Mg atoms.

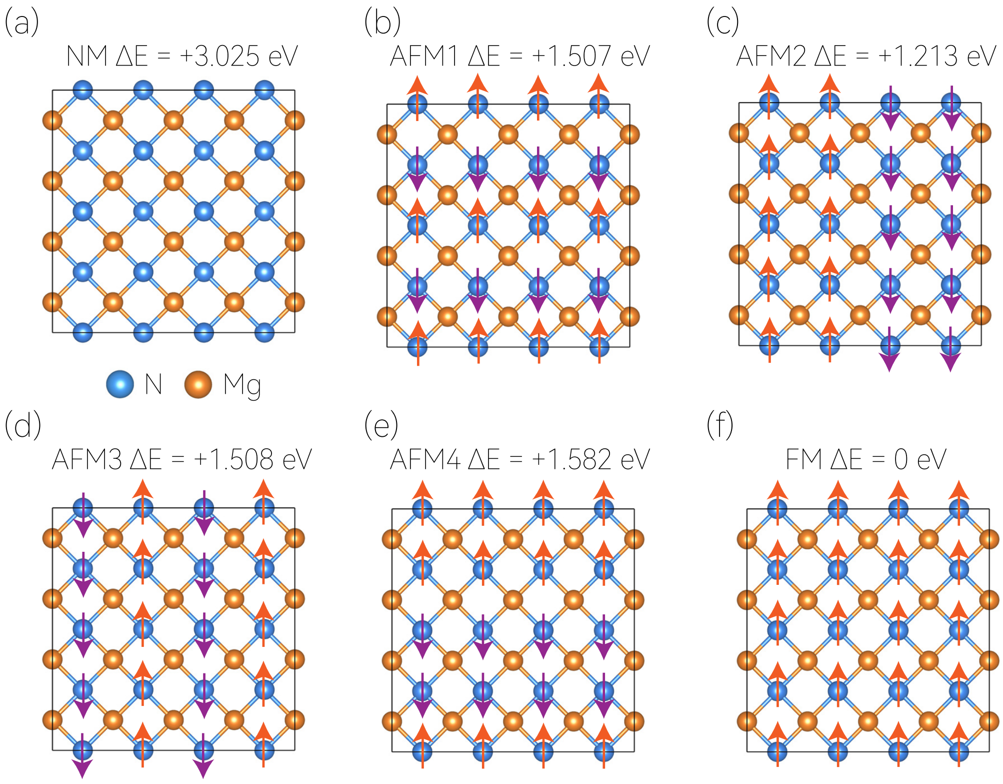

FIG. S2. Relative energies for (a) NM, (b)-(e) four AFM, and (f) FM states in $2 \times 2 \times 1$ supercell of Mg_4N_4. The arrows only indicate the spin directions of the N atoms. Key: blue, N atoms; orange, Mg atoms.

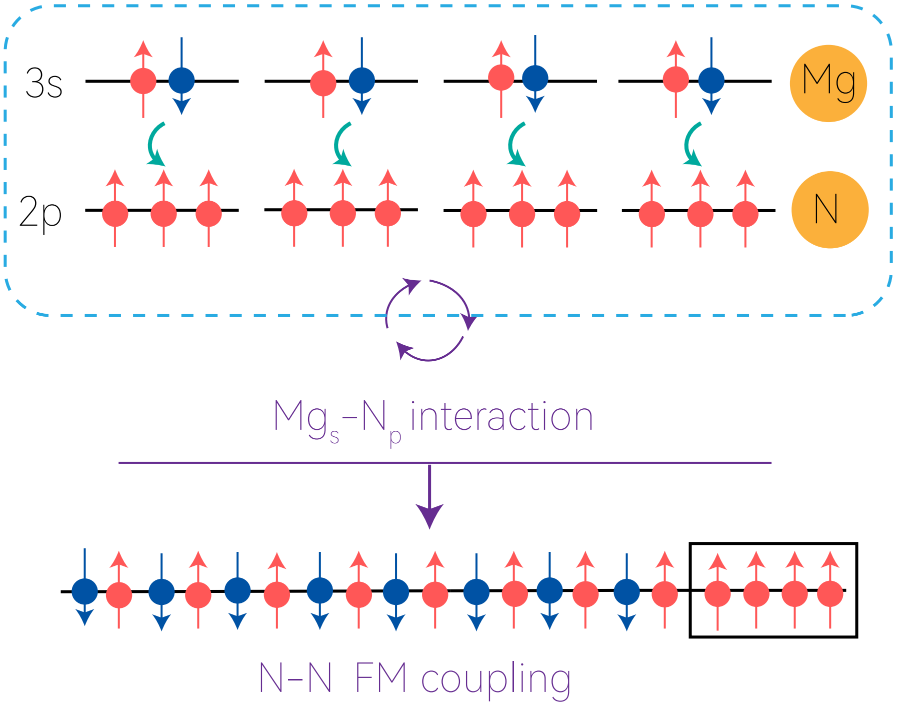

FIG. S3. Schematic diagrams for the origin of magnetic moment for N atoms within the Mg_4N_4 unit cell.

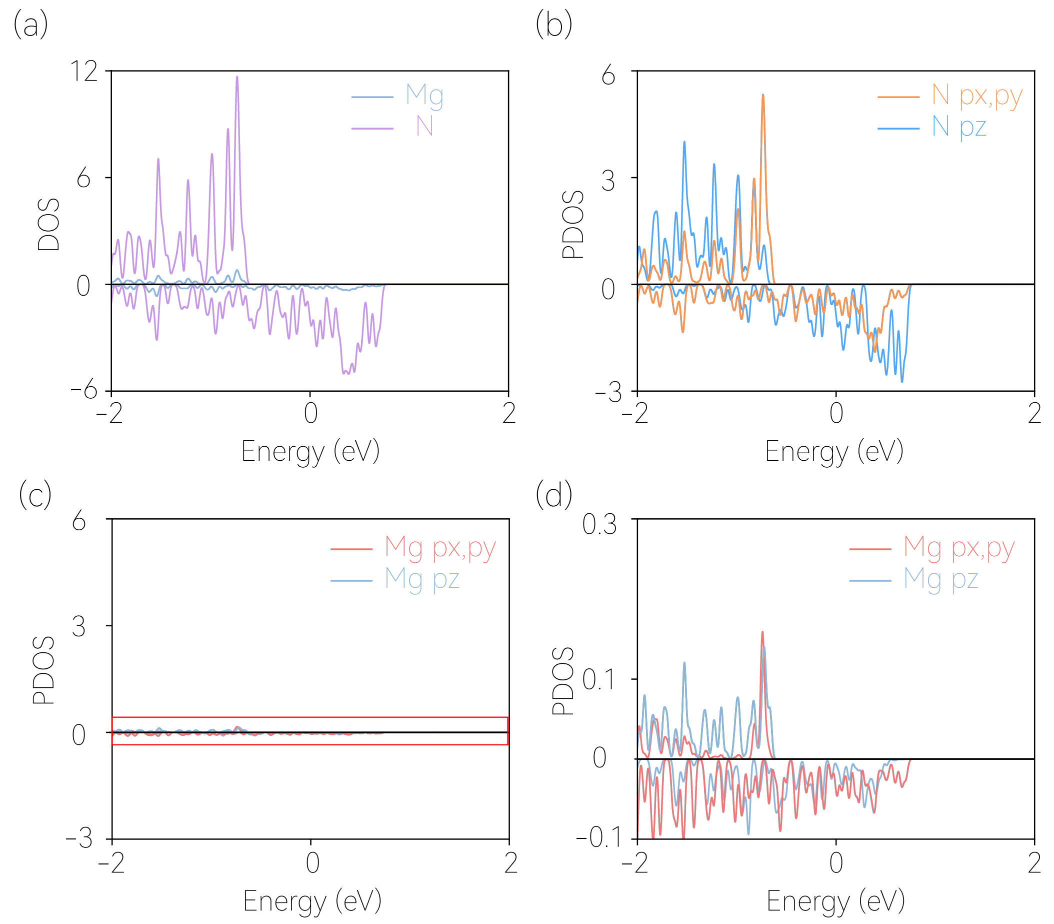

FIG. S4. (a)-(c) Projected density of states for Mg_4N_4. One can find that the Fermi level for Mg_4N_4 is dominated by N-p spin-down electronic orbitals. (d) is the enlarged figure of the red box region in (c).

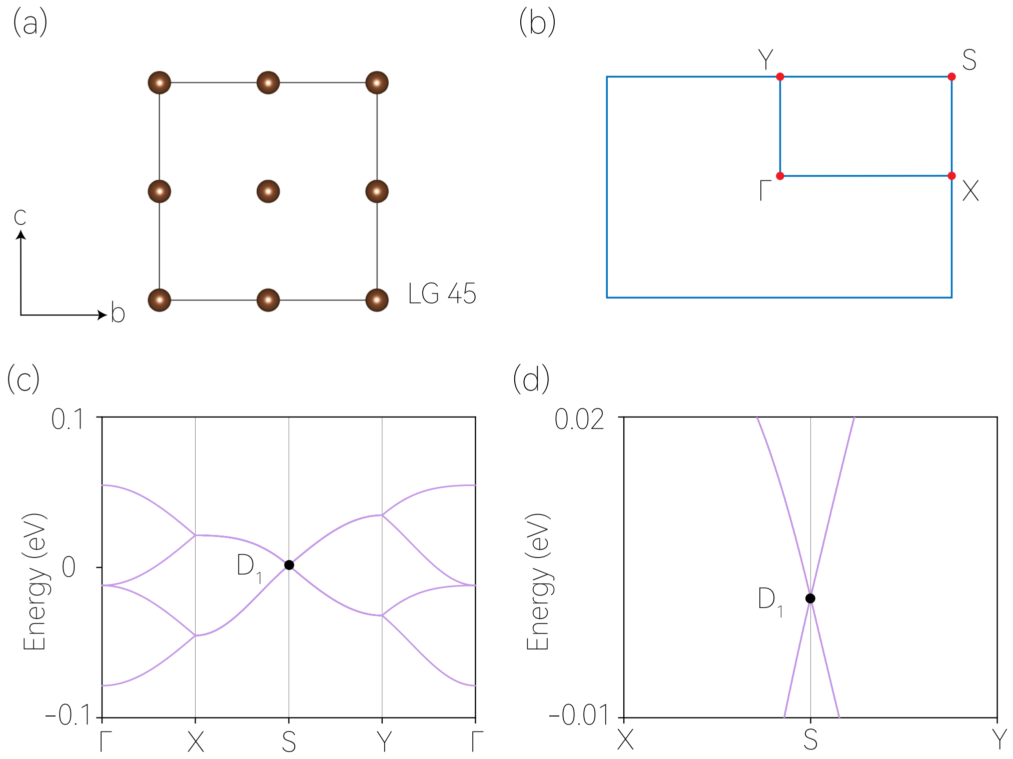

FIG. S5. (a) Spinless TB model with LG 45. (b) The 2D BZ and selected high-symmetry paths. (c) Calculated band structure for the spinless lattice model with LG 45. The Dirac point, D_1, location at the S high-symmetry point, is highlighted by a dot. (d) Enlarged band structure around the Dirac point (marked by a dot).

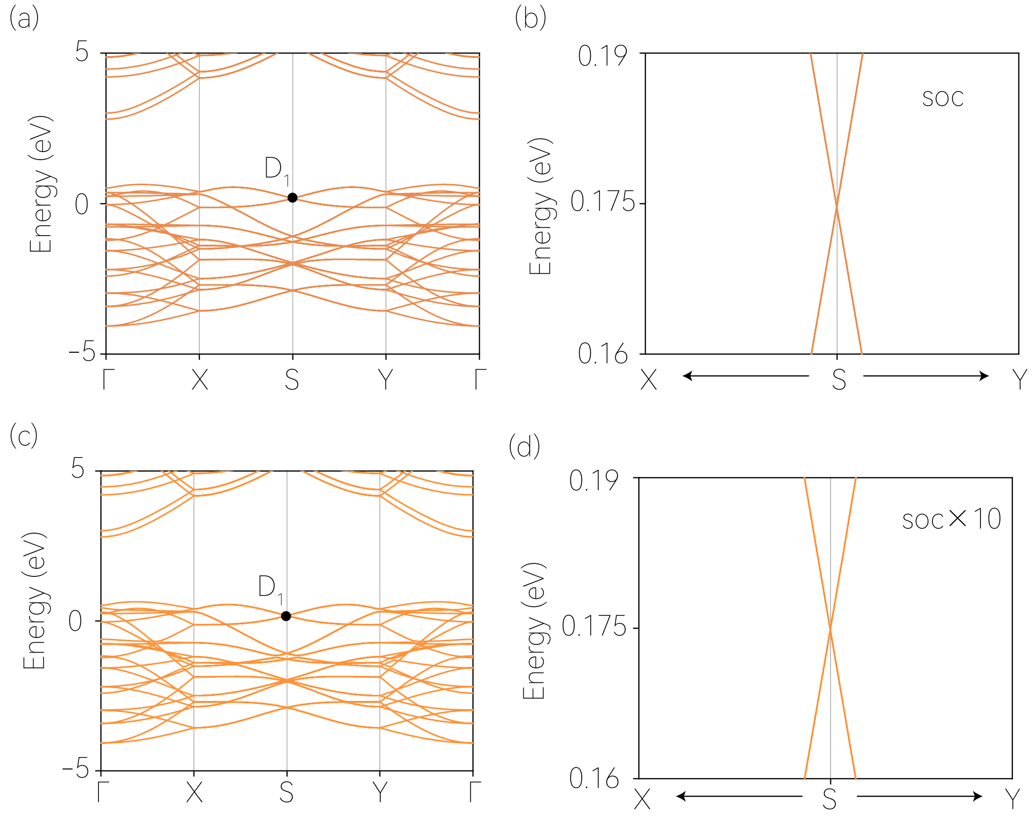

FIG. S6. (a) Band structure of 2D Mg_4N_4 with SOC and its enlarged bands around D_1 point (see (b)). (c) Band structure of 2D Mg_4N_4 with SOC $\times$ 10 and its enlarged bands around D_1 point (see (d)).

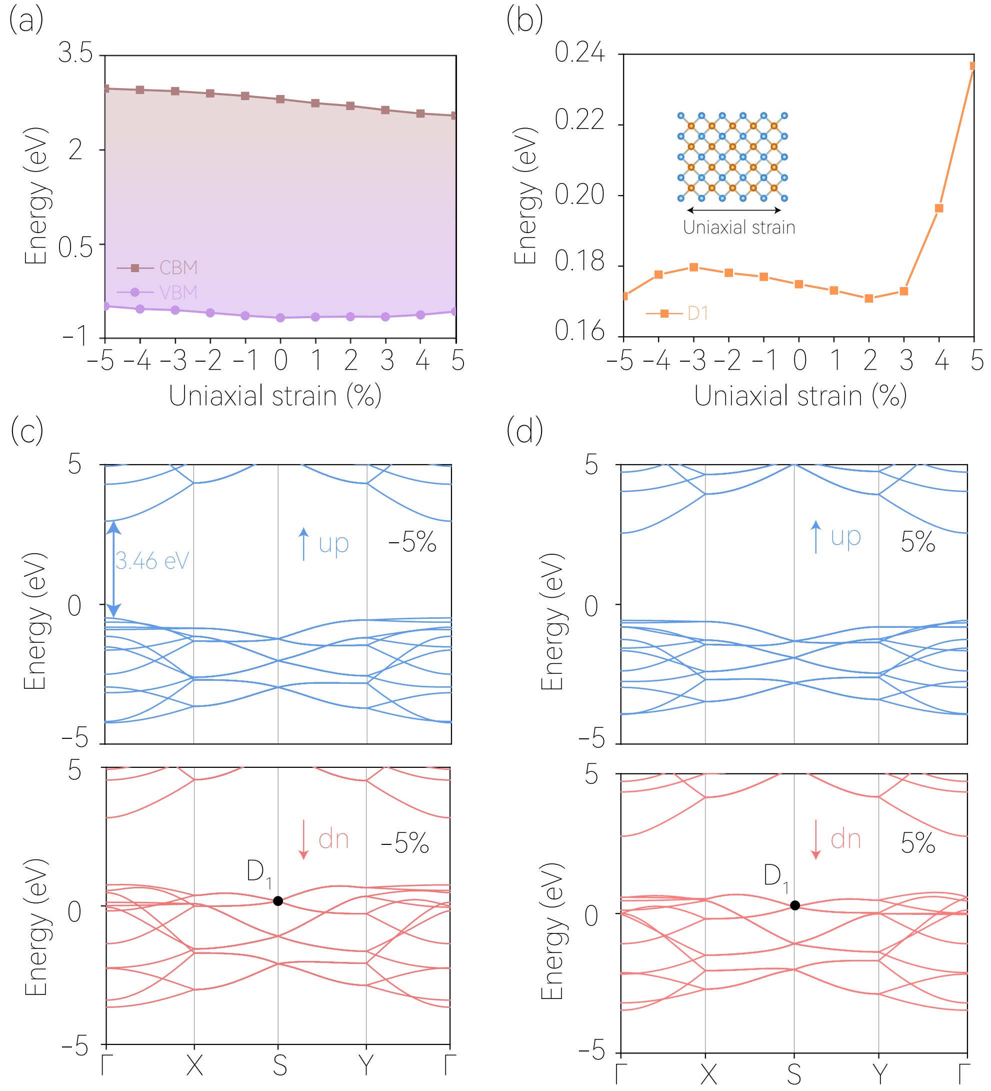

FIG. S7. (a) In the spin-up channel, the changing of the valence-band maximum (VBM) and conduction-band minimum (CBM) under -5%-5% uniaxial strains. The area formed by the curve shows the band gap in the spin-up channel. (b) In the spin-down channel, the changing of the Dirac point (D1) positions under -5%-5% uniaxial strains. (c) and (d) The band structures of 2D Mg_4N_4 in the spin-up and spin-down channels under -5% and +5% uniaxial strains.

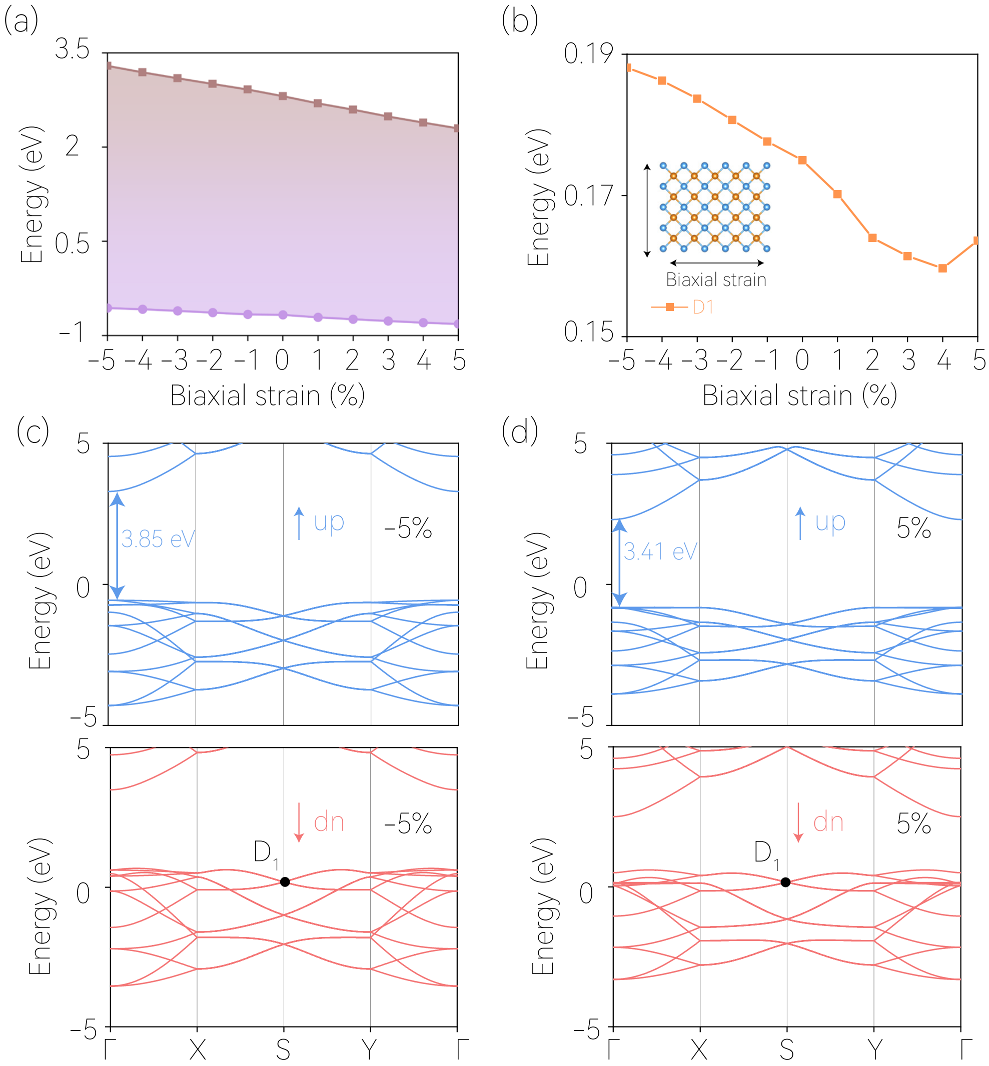

FIG. S8. (a) In the spin-up channel, the changing of the VBM and CBM under -5%-5% biaxial strains. The area formed by the curve shows the band gap in the spin-up channel. (b) In the spin-down channel, the changing of the Dirac point (D1) positions under -5%-5% biaxial strains. (c) and (d) The band structures of 2D Mg_4N_4 in the spin-up and spin-down channels under -5% and +5% biaxial strains.

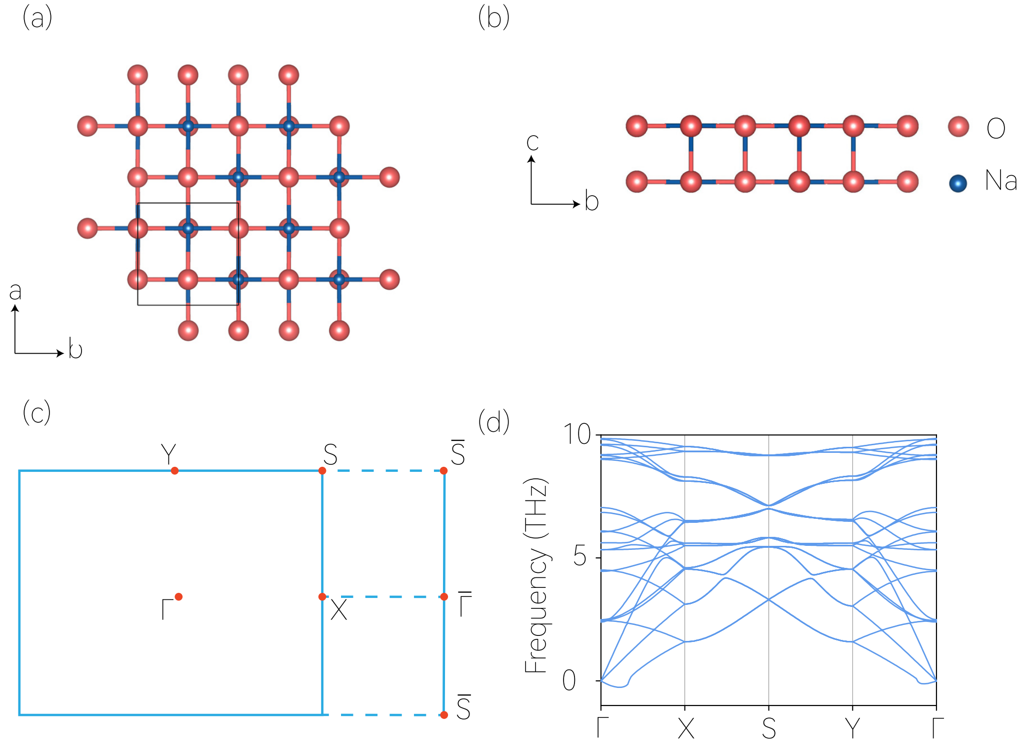

FIG. S9. (a) and (b) Top and side views of Na_4O_4 monolayer. The dashed box marks the unit cell. (c) 2D BZ and its projection to the [100] edge. (d) The calculated phonon dispersion for 2D Na_4O_4 unit cell. To obtain the force constants, we adopt a 2 × 2 × 1 supercell of Na_4O_4.

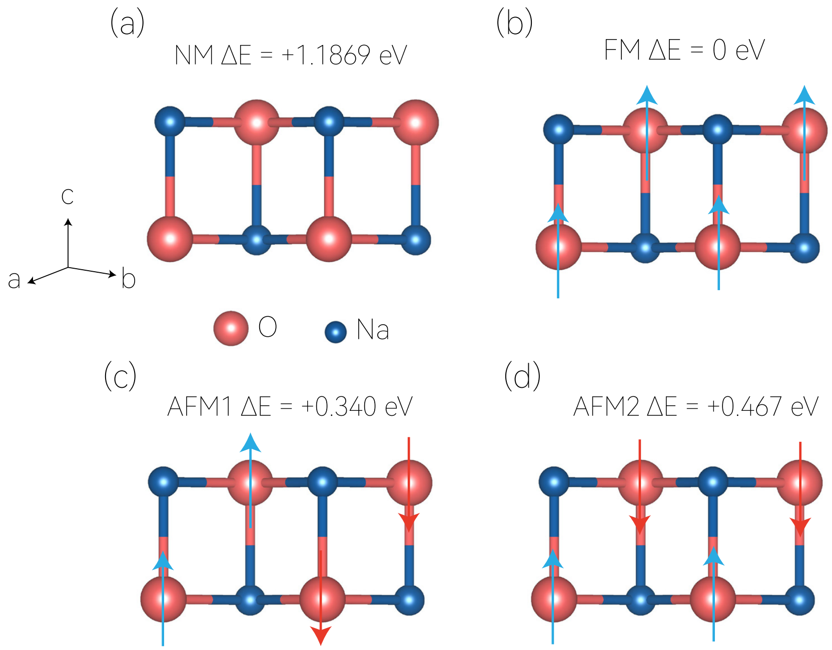

FIG. S10. Relative energies for (a) nonmagnetic (NM), (b) ferromagnetic (FM), and (c) and (d) two antiferromagnetic (AFM) states in a unit cell of Na_4O_4. The arrows only indicate the spin directions of the O atoms. Key: blue, Na atoms; red, O atoms.

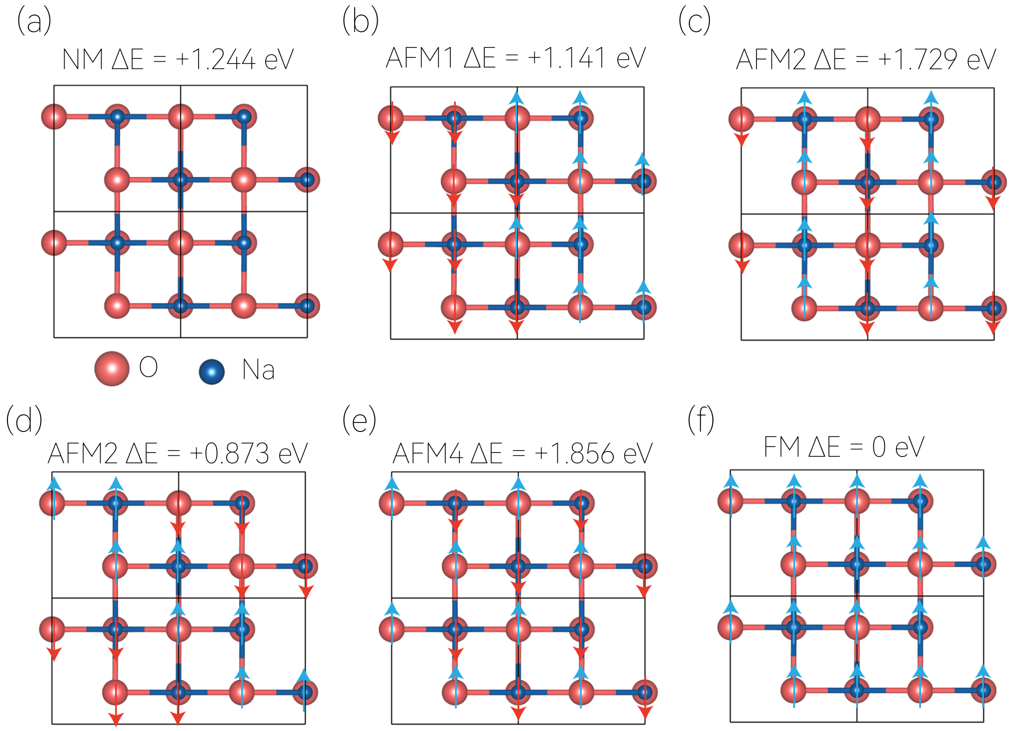

FIG. S11. Relative energies for (a) NM, (b)-(e) four AFM, and (f) FM states in 2 × 2 × 1 supercell of Na_4O_4. The arrows only indicate the spin directions of the O atoms. Key: blue, Na atoms; red, O atoms.

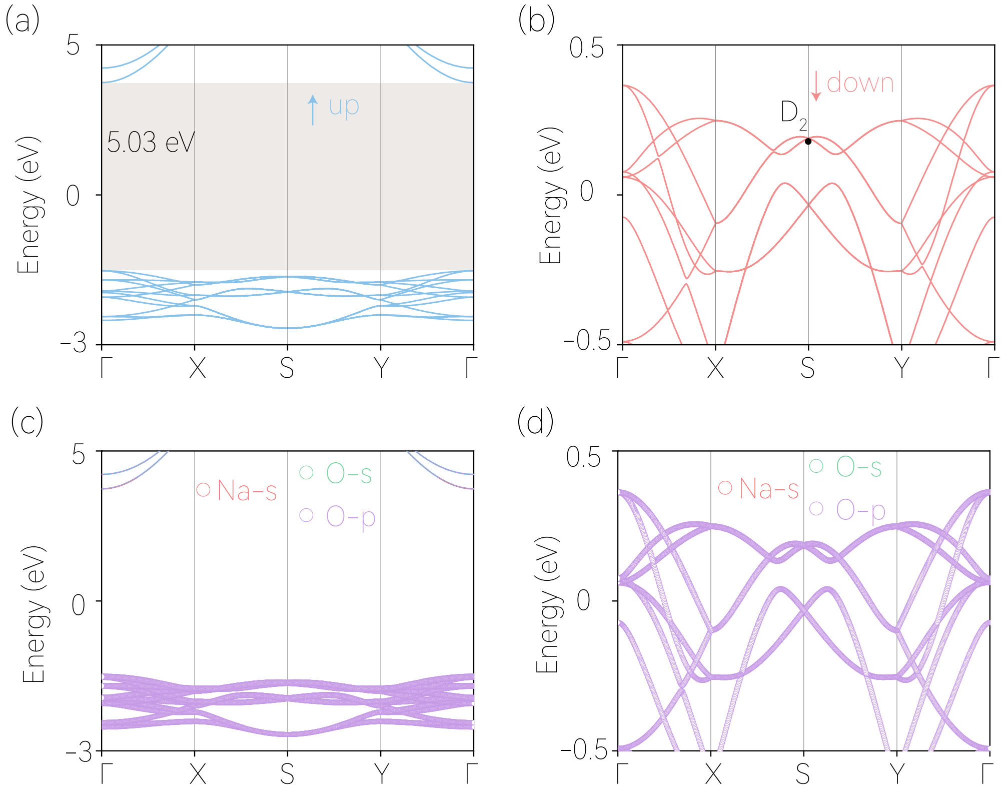

FIG. S12. (a) and (b) spin-up and spin-down band structures for Na_4O_4. The GGA method is used to obtain the band structures. (c) and (d) orbital-resolved band structures for Na_4O_4 in both spin channels.

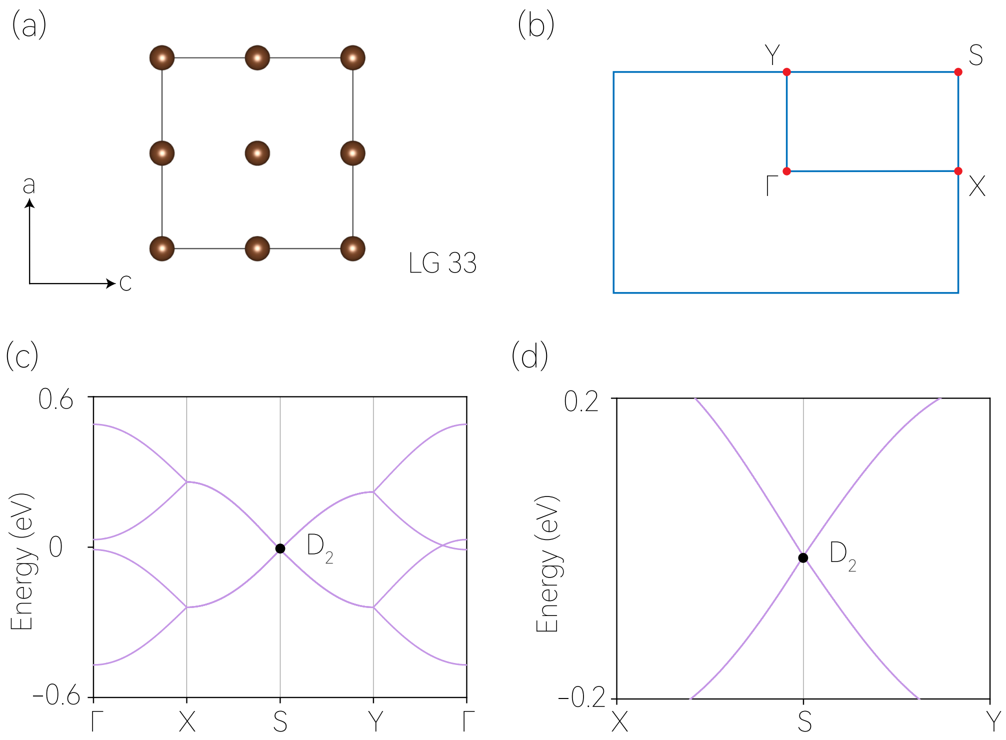

FIG. S13. (a) Spinless TB model with LG 33. (b) The 2D BZ and selected high-symmetry paths. (c) Calculated band structure for the spinless lattice model with LG 33. The Dirac point, D_2, location at the S high-symmetry point, is highlighted by a dot. (d) Enlarged band structure around the Dirac point D_2 (marked by a dot).

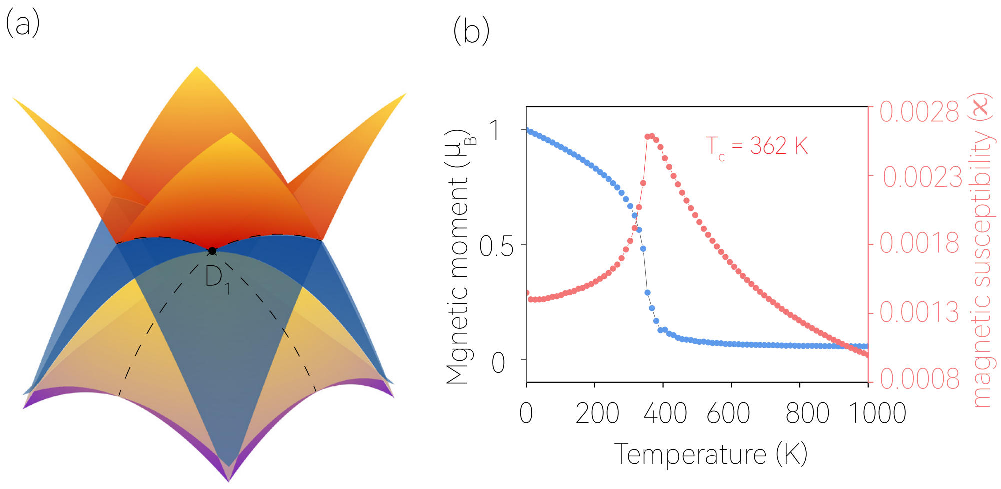

FIG. S14. (a) The 3D plot of the four bands around the D_2 Dirac point at the S high-symmetry point. (b) Dependence of magnetic moment and magnetic susceptibility on the temperature by the Heisenberg model via MC simulation.

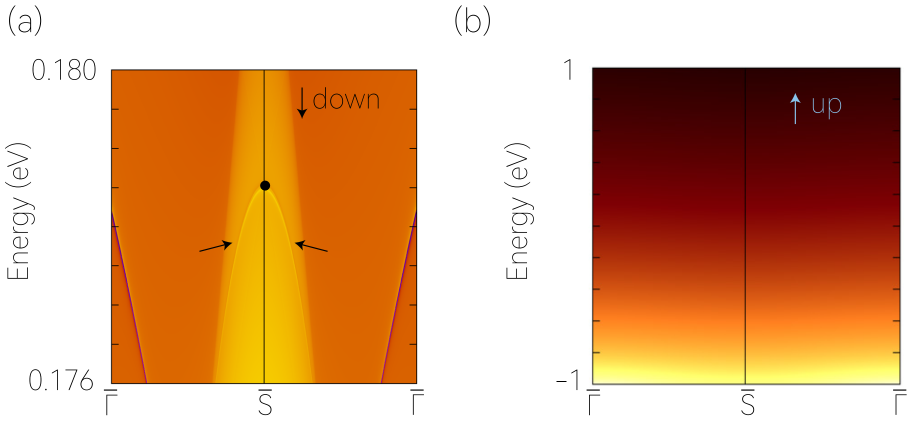

FIG. S15. [100] projective spectra in the spin-down (a) and spin-up (b) channels. From (a), the fully spin-polarized edge states, connecting to the projection of the D_2, are apparent.

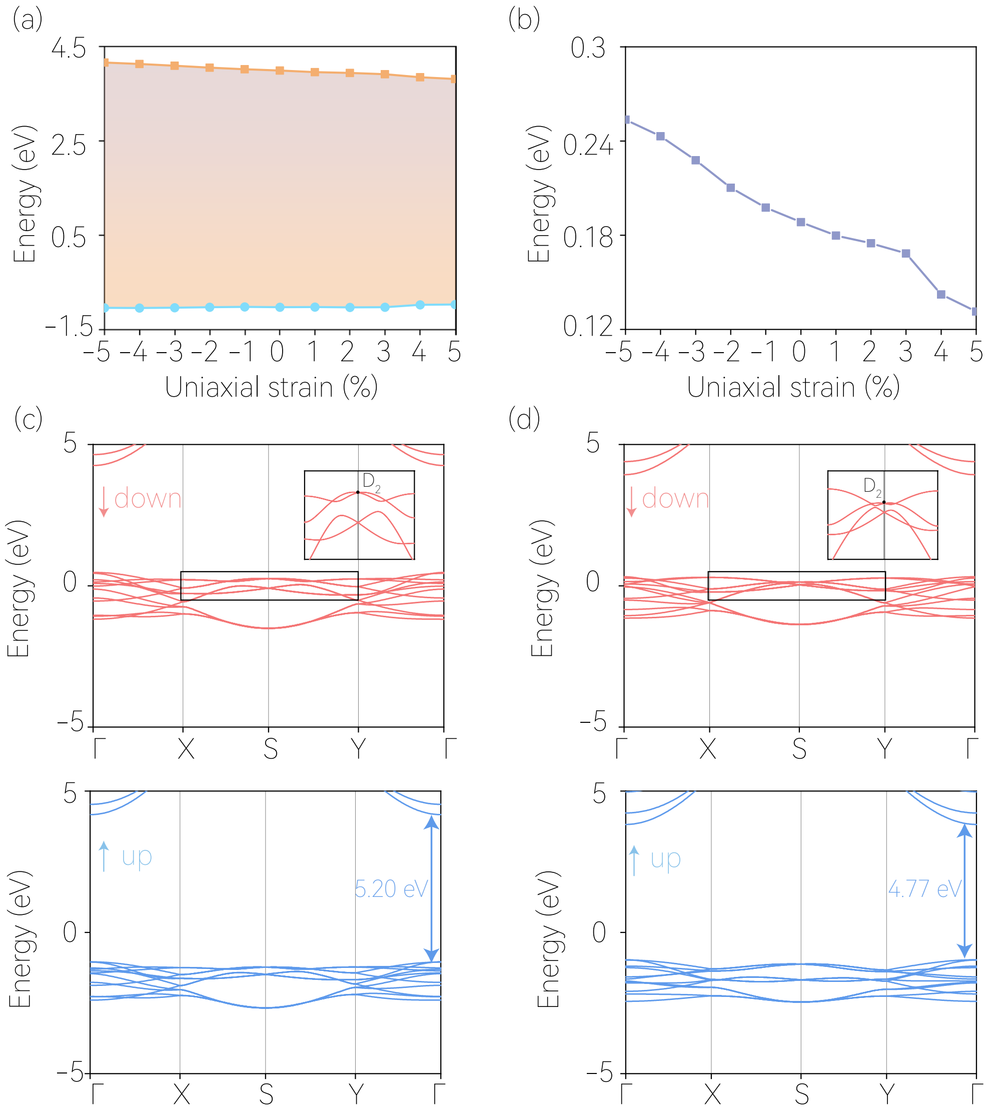

FIG. S16. (a) In the spin-up channel, the changing of the VBM and CBM under -5%-5% uniaxial strains. The area formed by the curve shows the band gap in the spin-up channel. (b) In the spin-down channel, the changing of the Dirac point (D2) positions under -5%-5% uniaxial strains. (c) and (d) The band structures of 2D Na_4O_4 in the spin-up and spin-down channels under -5% and +5% uniaxial strains, respectively.

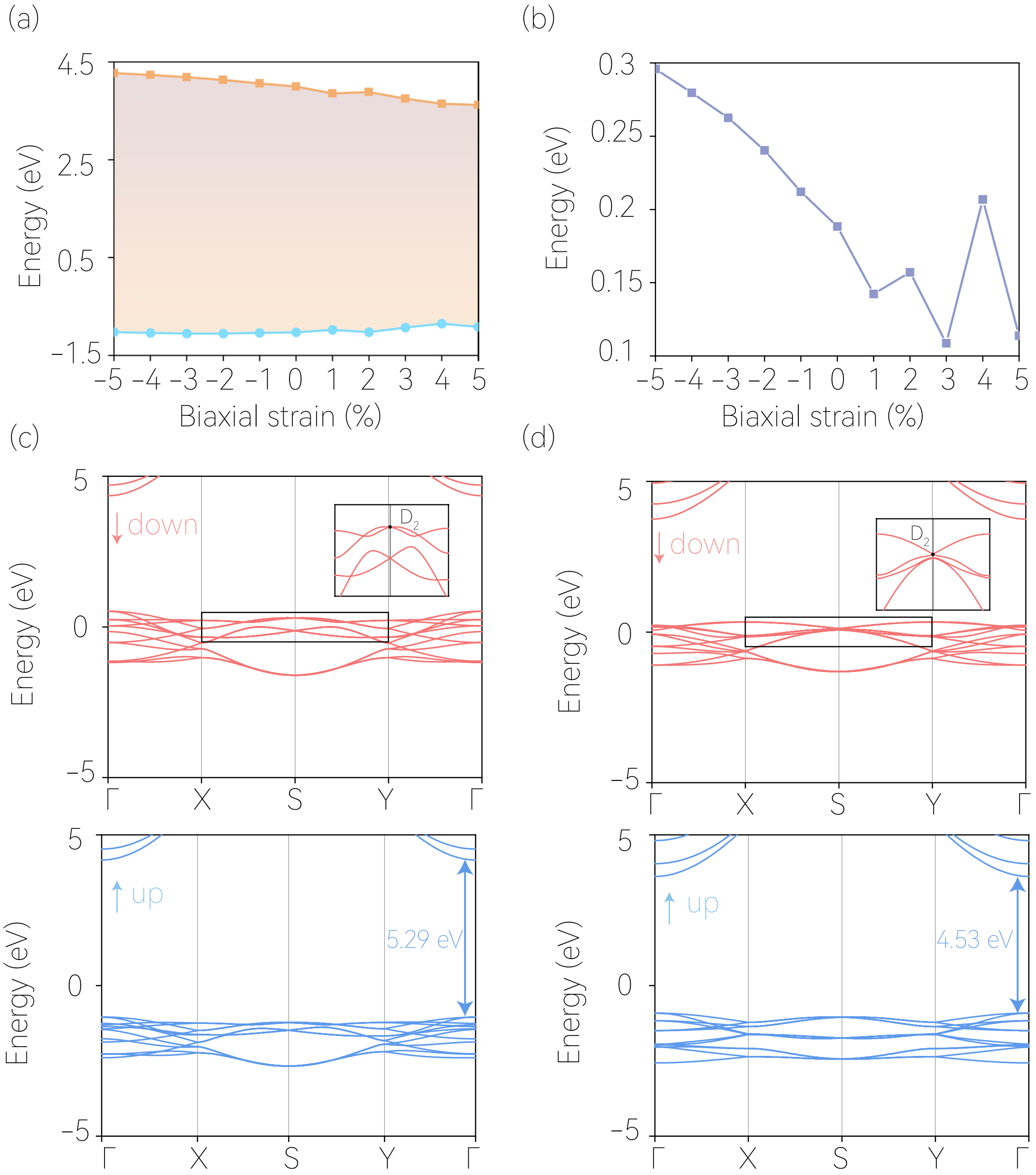

FIG. S17. (a) In the spin-up channel, the changing of the VBM and CBM under -5%-5% biaxial strains. The area formed by the curve shows the band gap in the spin-up channel. (b) In the spin-down channel, the changing of the Dirac point (D_2) positions under -5%-5% biaxial strains. (c) and (d) The band structures of 2D Na_4O_4 in the spin-up and spin-down channels under -5% and +5% biaxial strains, respectively.

TABLE S2. Atomic positions for Mg and N atoms in the Mg_4N_4 monolayer.

Atom	Position
Mg1	(0.000 0.750 0.500)
Mg2	(0.000 0.250 0.500)
Mg3	(0.500 0.250 0.500)
Mg4	(0.500 0.750 0.500)
N1	(0.250 1.000 0.522)
N2	(0.750 0.000 0.478)
N3	(0.250 0.500 0.478)
N4	(0.750 0.500 0.522)

TABLE S3. Total and atomic magnetic moments for Mg_4N_4 monolayer.

Atoms	$Mg_{(1-4)}$	$N_{(5-8)}$	Total
$M_t(\mu_B)$	0	0.65	2.60

TABLE S4. Simulated values of synthesized CrX_3 (X = Cl, Br, and I) monolayers. Clearly, our simulated $T_c{}^{MC}$ (K) is consistent with the experimentally measured $T_c{}^{Exp}$ (K) for all CrX_3 monolayers.

Monolayer	a (Å)	J (meV)	$T_c{}^{MC}$ (K)	$T_c{}^{Exp}$ (K)
$CrCl_3$	6.12	0.566	16	17
$CrBr_3$	6.50	0.722	32	37
CrI_3	7.08	0.907	47	45

TABLE S5. Relative energies of FM[100], FM[001], and FM[010] states for Mg_4N_4 monolayer. SOC is added.

	100	001	010
Energy (meV)	+0.00539	0	+0.00330

TABLE S6. Atomic positions for Na and O atoms in the Na_4O_4 monolayer.

Atom	Position
Na1	(0.999 0.252 0.549)
Na2	(0.000 0.752 0.451)
Na3	(0.501 0.752 0.549)
Na4	(0.999 0.252 0.451)
O1	(0.999 0.252 0.452)
O2	(0.001 0.752 0.548)
O3	(0.501 0.752 0.452)
O4	(0.499 0.252 0.548)

TABLE S7. Total and atomic magnetic moments for Na_4O_4 monolayer.

Atoms	$Na_{(1-4)}$	$O_{(5-8)}$	Total
$M_t(\mu_B)$	0	0.81	3.24
